# Mitogenome evolution in the last surviving woolly mammoth population reveals neutral and functional consequences of small population size

**DOI:** 10.1002/evl3.33

**Published:** 2017-11-28

**Authors:** Patrícia Pečnerová, Eleftheria Palkopoulou, Christopher W. Wheat, Pontus Skoglund, Sergey Vartanyan, Alexei Tikhonov, Pavel Nikolskiy, Johannes van der Plicht, David Díez‐del‐Molino, Love Dalén

**Affiliations:** ^1^ Department of Bioinformatics and Genetics Swedish Museum of Natural History Stockholm Sweden; ^2^ Department of Zoology Stockholm University Stockholm Sweden; ^3^ Department of Genetics Harvard Medical School Boston Massachusetts 02115; ^4^ Broad Institute of Harvard and MIT Cambridge Massachusetts 02142; ^5^ North‐East Interdisciplinary Scientific Research Institute N.A.N.A. Shilo Far East Branch, Russian Academy of Sciences (NEISRI FEB RAS) Magadan Russia; ^6^ Zoological Institute of Russian Academy of Sciences Saint‐Petersburg Russia; ^7^ Institute of Applied Ecology of the North North‐Eastern Federal University Yakutsk Russia; ^8^ Geological Institute of the Russian Academy of Sciences Moscow Russia; ^9^ Centre for Isotope Research Groningen University Groningen The Netherlands; ^10^ Faculty of Archaeology Leiden University Leiden The Netherlands

**Keywords:** *Mammuthus primigenius*, mitochondrial genomes, woolly mammoth, Wrangel Island

## Abstract

The onset of the Holocene was associated with a global temperature increase, which led to a rise in sea levels and isolation of the last surviving population of woolly mammoths on Wrangel Island. Understanding what happened with the population's genetic diversity at the time of the isolation and during the ensuing 6000 years can help clarify the effects of bottlenecks and subsequent limited population sizes in species approaching extinction. Previous genetic studies have highlighted questions about how the Holocene Wrangel population was established and how the isolation event affected genetic diversity. Here, we generated high‐quality mitogenomes from 21 radiocarbon‐dated woolly mammoths to compare the ancestral large and genetically diverse Late Pleistocene Siberian population and the small Holocene Wrangel population. Our results indicate that mitogenome diversity was reduced to one single haplotype at the time of the isolation, and thus that the Holocene Wrangel Island population was established by a single maternal lineage. Moreover, we show that the ensuing small effective population size coincided with fixation of a nonsynonymous mutation, and a comparative analysis of mutation rates suggests that the evolutionary rate was accelerated in the Holocene population. These results suggest that isolation on Wrangel Island led to an increase in the frequency of deleterious genetic variation, and thus are consistent with the hypothesis that strong genetic drift in small populations leads to purifying selection being less effective in removing deleterious mutations.

**Impact Summary**

While most of the Pleistocene megafauna species became extinct at the end of the last ice age, the woolly mammoth survived in small insular populations, most notably on Wrangel Island where it survived until 4000 years before present. Genetic data suggest that compared to the large and diverse Pleistocene population, Holocene mammoths on Wrangel Island had low genetic diversity. However, it is still unclear to what extent genetic diversity was lost as a consequence of a founder effect when rising sea levels led to the formation of the island, compared to the subsequent effect of small effective population size during the ensuing 6000 years. To examine this, we sequenced mammoth mitogenomes from before and after the isolation on Wrangel Island. Our results show a severe loss in genetic diversity and fixation of a mutation with potential functional consequences at the time the population was established, supporting the hypothesis of a founder effect. However, the observation of an increase in the evolutionary rate following isolation on the island is consistent with an elevated impact of genetic drift leading to purifying selection becoming less efficient. Our findings add some details into the mosaic of complex processes that preceded the woolly mammoth's extinction and serve as a rare example of testing basic population genetic concepts in a wild population.

The fate of taxa during periods of climate change can be simplified into three processes: adaptation, migration, or extinction (Davis and Shaw 2001; Aitken et al. 2008). Changes in environmental conditions can potentially lead to adaptation through selection on standing genetic variation. However, if these changes are too rapid, or the amount of genetic variation is insufficient, species may need to track geographical changes in habitat availability to persist. The woolly mammoth (*Mammuthus primigenius*) inhabited the Northern Hemisphere for ∼800 thousand years (kyr), from the late Middle Pleistocene to early Holocene (Lister and Sher 2001). Mammoths thus survived a number of alternating glacials and interglacials, but went extinct during the Holocene interglacial period ∼4 thousand years before present (kyr calBP) (Vartanyan et al. 1995). Even though mammoths were originally thought to have become extinct at the Pleistocene/Holocene boundary (∼11,700 yr calBP), along with other representatives of the Pleistocene megafauna in a phenomenon known as the *Quaternary extinction event* (Stuart 1991), mammoth remains with radiocarbon ages as young as 4 kyr calBP have been found on Wrangel Island (Vartanyan et al. 1993; Stuart et al. 2002). Wrangel Island thus represents the refugium of the last surviving population of the species.

Wrangel Island became separated from continental Siberia ∼10 kyr calBP following the rise in sea levels after the Last Glacial Maximum (LGM) (Vartanyan et al. 2008; Arppe et al. 2009). During the Late Pleistocene, Wrangel formed a mountainous area in the otherwise flat Beringian landscape, with parts covered by an inactive rock glacier and perennial snowfields (Vartanyan et al. 2008). The fossil record and strontium isotopes in bones indicate that mammoths were not permanent residents in the region during the Late Pleistocene, but rather visited (what later became) Wrangel Island during seasonal migrations (Vartanyan et al. 2008; Arppe et al. 2009).

After the isolation, however, mammoths were confined to and survived on Wrangel Island for an additional ∼6 kyr. The process leading up to the mammoth's extinction has been studied using both nuclear and mitochondrial DNA. Initial studies on short mitochondrial sequences (Nyström et al. 2010) and microsatellite loci (Nyström et al. 2012) suggested that, after an abrupt loss of genetic variation related to the isolation event, genetic diversity was retained or even slightly increased during the subsequent ∼6 kyr. More recent analyses of two complete nuclear genomes revealed that the Wrangel mammoth genome – compared to a Pleistocene mainland mammoth genome – had lower observed heterozygosity and a higher proportion of the genome allocated in runs of homozygosity (ROH), which could have been either the result of a founder effect during the isolation of the island, and/or repeated breeding between distant relatives due to the small Holocene effective population size (Palkopoulou et al. 2015).

To further examine the genetic changes that took place during and after the isolation of Wrangel Island, we generated high‐quality mitogenomes from 21 woolly mammoths, including 14 Holocene Wrangel individuals (Fig. 1), and compared these with 21 previously published sequences. We used this data to test the hypothesis that the Wrangel Island population was established by a small number of individuals (i.e., a founder effect), as well as to characterize in situ evolution of neutral and functional mitochondrial variation in the small isolated Holocene population.

**Figure 1:**
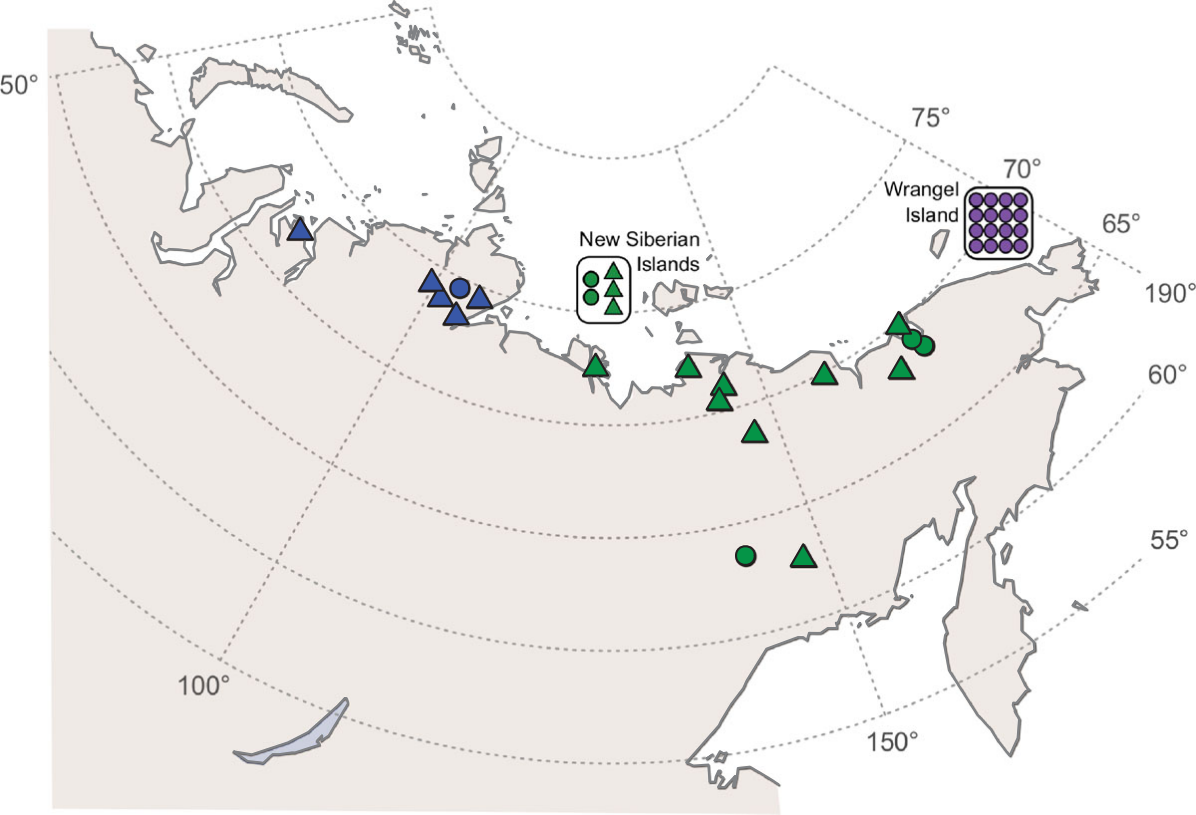
Map depicting the geographic origin of the samples, including 16 mitogenomes from Wrangel Island. Samples analyzed in this study are depicted as circles, while previously published samples are shown as triangles. Colors show the geographical classification used in this study: blue – Western Siberia, green – Central Siberia, and purple – Wrangel Island. The map was created using R (R Development Core Team 2016; available from https://www.R-project.org/).

## Methods

Fifty‐one woolly mammoth specimens collected on Wrangel Island (*n* = 35) and the Siberian mainland (*n* = 16) were analyzed in this study. The samples consisted of bones, tusks, and teeth, and those with unknown age were radiocarbon dated using Acceleration Mass Spectrometry (AMS) in Oxford and Groningen (Table 1). The results are reported in conventional radiocarbon years (BP), which includes correction for isotopic fractionation and usage of the conventional half‐life (Mook and van der Plicht 1999). The ^14^C dates were calibrated into calendar ages using the recommended calibration curve IntCal13 (Reimer et al. 2013) using the program OxCal 4.2 (Ramsey 2009). Medians of the calibrated dates are reported in calBP, that is calendar years relative to 1950 AD.

**Table 1:** Characteristics of mammoth samples used in this study, including the estimated haplotypes (W = Wrangel, S = Siberia)

Lab ID	^14^C Lab no.	^14^C BP date ± error	Median CalBP	Material	Location	Endogenous DNA (%)	Average fragment length (bp)	Accession no.	Reference	Average Coverage	Haplotype
E469D	Ua‐13366	3685 ± 60	4024	Tooth	Wrangel Island	29.59	64.6	MG334270	This study	9.5	W7
E468	LU‐2741	3730 ± 40	4079	Tusk	Wrangel Island	16.23	101.5	MG334269	This study	31.9	W1
E467	AA40665	3905 ± 47	4336	Tooth	Wrangel Island	80.35	81.9	MG334268	This study	11.7	W1
E466	GIN‐6985	3920 ± 40	4354	Tusk	Wrangel Island	17.68	88.9	MG334267	This study	8.8	W6
E465	LU‐4448	4120 ± 110	4643	Tusk	Wrangel Island	12.23	98.8	MG334266	This study	38.2	W5
E464	Ua‐13375	4210 ± 70	4726	Tooth	Wrangel Island	70.55	84.2	MG334265	This study	21.8	W1
E460	LU‐2756	4400 ± 40	4969	Tusk	Wrangel Island	87.13	90.1	MG334264	This study	39.0	W4
M28	GIN‐6988	5610 ± 40	6380	Tusk	Wrangel Island	56.59	151.6	MG334281	This study	276.7	W4
L459	OxA‐30117*	6148 ± 32	7060	Tusk	Wrangel Island	0.47	77.8	MG334276	This study	5.5	W4
M26	LU‐2799	6260 ± 50	7194	Tooth	Wrangel Island	27.69	71.2	MG334280	This study	25.8	W3
L386	Ua‐13374	6410 ± 90	7336	Tooth	Wrangel Island	82.37	73.0	MG334274	This study	12.0	W4
M23	LU‐4449	6560 ± 60	7470	Tusk	Wrangel Island	75.85	111.5	MG334279	This study	170.9	W2
M17	Ua‐13372	7510 ± 80	8318	Tooth	Wrangel Island	68.16	71.2	MG334278	This study	27.2	W1
L468	OxA‐30122*	7711 ± 36	8491	Bone	Wrangel Island	15.30	70.8	MG334277	This study	7.8	W1
P011	GrA‐ 65691*	10,240 ± 50	11972	Tusk	Taimyr Peninsula	2.11	66.6	MG334285	This study	5.5	S5
P005	GrA‐ 65686*	10,920 ± 50	12775	Tusk	New Siberian Islands	91.70	75.7	MG334283	This study	10.8	S3
Ber28	UCIAMS38670	12,125 ± 30	14011	n.a.	Berelekh	n.a.	n.a.	KX027495	Enk et al. 2016		S24
Krause	KIA‐25289	12,170 ± 50	14056	Bone	Yakutia	n.a.	n.a.	DQ188829	Krause et al. 2006		S22
L410	OxA‐31180*	12,370 ± 55	14408	Tooth	Wrangel Island	29.31	80.0	MG334275	This study	6.8	W9
L158	OxA‐20046	12,380 ± 45	14431	Humerus	Pioneyveem River, Chukotka	47.91	76.7	MG334272	This study	17.7	S1
P009	GrA‐65689*	13,030 ± 60	15602	Tusk	New Siberian Islands	82.40	67	MG334284	This study	6.8	S4
L164	OxA‐20048	13,935 ± 50	16901	tusk	Pioneyveem River, Chukotka	85.92	83.9	MG334273	This study	9.8	S2
GilbertM15	OxA‐19605	13,995 ± 55	16996	Hair	Ayon Island, Chukotka	n.a.	n.a.	EU153446	Gilbert et al. 2008		S13
GilbertM18	OxA‐17116	17,125 ± 70	20655	Hair	Gydan Peninsula	n.a.	n.a.	EU153447	Gilbert et al. 2007		S14
GilbertM4	OxA‐17098	18,545 ± 70	22422	Hair	n.a.	n.a.	n.a.	EU153456	Gilbert et al. 2007		S9
GilbertM19	GrN‐28258	18,560 ± 50	22434	Hair	Yakutia	n.a.	n.a.	EU153448	Gilbert et al. 2008		S9
GilbertM2	UtC‐8138	20,380 ± 140	24507	Hair	Taimyr Peninsula	n.a.	n.a.	EU153449	Gilbert et al. 2007		S7
GilbertM3	Beta‐148647	20,620 ± 70	24833	Hair	Taimyr Peninsula	n.a.	n.a.	EU153455	Gilbert et al. 2007		S8
GilbertM26	OxA‐17114	24,740 ± 110	28769	Hair	Indigirka	n.a.	n.a.	EU153454	Gilbert et al. 2007		S19
Poinar	Beta‐210777	27,740 ± 220	31501	Bone	Taimyr Peninsula	n.a.	n.a.	EU155210	Poinar et al. 2006		S20
GilbertM13	T‐171	35,800 ± 1200	40418	Hair	Lena River	n.a.	n.a.	EU153445	Gilbert et al. 2007		S12
E470	LU‐3511	37,080 ± 1650	41632	Bone	Wrangel Island	31.72	73.4	MG334271	This study	11.5	W8
Oimyakon	GrA‐30727	41,300 ± 900	44828	Skin	Yakutia	n.a.	n.a.	MG334282	Palkopoulou et al. 2015		S23
GilbertM8	OxA‐17102	46,900 ± 700	46962	Hair	Magadan	n.a.	n.a.	EU153458	Gilbert et al. 2007		S11
GilbertM22	OxA‐17111	50,200 ± 900	50304	Hair	New Siberian Islands	n.a.	n.a.	EU153452	Gilbert et al. 2007		S17
GilbertM25	OxA‐19610	59,300 ± 2700	60495	Hair	Yakutia	n.a.	n.a.	EU153453	Gilbert et al. 2008		S18
GilbertM1	n.a.	n.a.	n.a.	Hair	n.a.	n.a.	n.a.	EU153444	Gilbert et al. 2007		S6
GilbertM5	n.a.	n.a.	n.a.	Hair	n.a.	n.a.	n.a.	EU153457	Gilbert et al. 2007		S10
GilbertM20	OxA‐19608	>63,500	n.a.	Hair	New Siberian Islands	n.a.	n.a.	EU153450	Gilbert et al. 2008		S15
GilbertM21	OxA‐19609	>58,000	n.a.	Hair	New Siberian Islands	n.a.	n.a.	EU153451	Gilbert et al. 2008		S16
Rogaev	MAG‐1000	33,750—31,950	n.a.	Muscle	Enmynveem River, Chukotka	n.a.	n.a.	DQ316067	Rogaev et al. 2006		S21
2002/472	UCIAMS38677	>48,800	n.a.	n.a.	Taimyr Peninsula	n.a.	n.a.	KX027489	Enk et al. 2016		S25

Asterisks indicate new radiocarbon dates; n.a. = not available.

### DATA PREPARATION

Sample E469D was extracted using a method optimized for highly degraded samples (Dabney et al. 2013), while sequence data for samples labeled “E” come from (Palkopoulou et al. 2015), but with new consensus sequences generated in this study. Samples labeled “L,” “M,” and “P” were extracted according to protocol C in Yang et al. (1998) as modified in Brace et al. (2012). Double stranded Illumina libraries were prepared from 20 μL of DNA extract according to Meyer and Kircher (2010), using uracil‐treatment with the USER enzyme (New England Biolabs; Briggs et al. 2010). During the blunt‐end repair, USER enzyme was added so that the final concentration was 0.15 U/μL in the reaction mix described in “Step 4” of Meyer and Kircher (2010). T4 DNA polymerase was added to the reaction mix following a three‐hour incubation at 37°C. Subsequently, blunt‐end repair incubation and all following steps were performed according to the protocol by Meyer and Kircher (2010). Indexing amplifications were prepared with AccuPrime™ Pfx DNA Polymerase (Life Technologies) using one indexing primer per library and the following amplification conditions: 95°C for 2 minutes and between 8 and 14 cycles of: 95°C for 15 seconds, 60°C for 30 seconds, 68°C for 30 seconds. Libraries were purified along with size selection using Agencourt AMPure XP beads (Beckman Coulter) targeting fragments between 100 and 500 base pairs to remove unligated adapters, primer dimers, and long contaminant sequences. Library concentrations were measured with a high‐sensitivity DNA chip on a Bioanalyzer 2100 (Agilent). Multiplexed libraries were pooled in two separate pools in equimolar concentrations and shotgun‐sequenced on two lanes of Illumina HiSeq2500 with a 2 × 125 bp setup in the HighOutput mode.

### DATA PROCESSING

Bcl to Fastq conversion was performed using bcl2Fastq 1.8.3 from the CASAVA software suite. SeqPrep 1.1 (https://github.com/jstjohn/SeqPrep) was used to trim adapters and merge paired‐end reads, using default settings and a minor modification to the source code, allowing us to choose the best quality scores of bases in the merged region instead of aggregating the scores (Palkopoulou et al. 2015). The modified file is available for download at the webpage http://www.palaeogenetics.com/adna/data.

Sequencing reads were processed with BWA 0.7.8 (Li and Durbin 2010) and SAMtools 0.1.19 (Li et al. 2009). Following Prufer et al. (2014), we modified the woolly mammoth mitochondrial genome (GenBank accession no. DQ188829) by copying the first 240 bp to the end of the sequence to facilitate mapping and to avoid lower coverage in the marginal parts of the sequence. This modified mitochondrial genome was merged with the African savanna elephant nuclear genome (LoxAfr4) generated by the Broad Institute, and the merged sequence was used as a reference to avoid mapping nuclear copies of mitochondrial DNA (numts) to the mitochondrial DNA reference genome (note, however, that this would not identify numts that have evolved in mammoths since their divergence from the African savanna elephant). Merged sequencing reads were mapped against the reference using the BWA aln algorithm with parameters adapted for ancient DNA reads that deactivate seeding (‐l 16500), allow more substitutions (‐n 0.01) and up to two gaps (‐o 2). BWA samse command was used to generate alignments. Reads mapping to the mitochondrial genome were extracted and processed in SAMtools 0.1.19 (Li et al. 2009), including converting the alignments in SAM format to BAM format, coordinate sorting, indexing, and removing duplicates (with the single‐end option “‐s”). Reads with mapping qualities below 20 were filtered out.

BAM files generated using SAMtools were uploaded to Geneious® 7.0.3 (Kearse et al. 2012) and consensus sequences were called for positions with at least 3X coverage using the majority rule, with ambiguous and low‐coverage positions called as undetermined.

To avoid incorrect consensus calling due to DNA damage characteristic for ancient samples, two steps were taken: (a) USER (Uracil‐Specific Excision Reagent) Enzyme was used to remove damaged sites, and (b) only positions covered by at least three bases, that is three individual replicates, were called.

Twenty‐one samples with consensus sequences resolved for at least 80% of positions of the mitogenome were aligned to 21 previously published mammoth mitogenomes (Table 1) in MAFFT 7.245 (Katoh and Standley 2013). The variable number tandem repeats (VNTR) section of the alignment was removed since it had not been assembled in the previously published data (Gilbert et al. 2007; Gilbert et al. 2008).

To verify that there was no bias introduced by using a clade I mammoth (Krause; DQ188829) as a reference, the data was also mapped against a clade II mammoth mitogenome (GilbertM25; EU153453). Despite only using a mitochondrial reference (rather than a merged nuclear‐mitochondrial reference as in the original processing) and omitting the “E” samples (Palkopoulou et al. 2015) from the analyses, the results were consistent and the haplotype network maintained the same structure, including the star‐like pattern of the Holocene haplotypes (Fig. S1).

### DEMOGRAPHIC AND PHYLOGENETIC ANALYSES

A median‐joining haplotype network of all mitogenomes was created in PopART (available at http://popart.otago.ac.nz). Sequences of mammoth mitochondrial clade I with finite radiocarbon dates were analyzed by Bayesian Inference in BEAST 1.8.0 (Drummond et al. 2012) using tip‐dating and the HKY+I substitution model, which was selected according to the Bayesian Information Criterion in ModelGenerator (Keane et al. 2006). A strict molecular clock was applied and mutation rate was estimated from the data, using a starting rate of 8.07 × 10^−8^ site^−1^ year^−1^ (Palkopoulou et al. 2013). To test for the extent of temporal signal in the data, we performed a date‐randomization test (Palkopoulou et al. 2013; Duchene et al. 2015). We used SiteSampler v1.1 (Ho and Lanfear 2010) to generate 20 data sets with randomly reassigned labels (i.e., dates) and we compared the substitution rate estimated by the randomized datasets to the estimate from real data.

Three different tree models were tested: constant size, Bayesian Skyline, and Bayesian Skyride. Constant size and Bayesian Skyride analyses were performed with default settings while the number of groups in the Bayesian Skyline model was adjusted to five to avoid overparametrization of the model (Drummond et al. 2005). To decide which model provides the best fit, we calculated the marginal likelihoods using path and stepping‐stone sampling as implemented in BEAST 1.8.0 (Baele et al. 2012; Baele et al. 2013). Bayes Factors were estimated using the marginal likelihoods, and we used the approach by Kass and Raftery (1995) to select the most appropriate model for further analyses (Table S1).

For all models, the Markov Chain Monte Carlo was set to run for 50 million generations, sampling every 5000^th^ generation. Information from the sampled trees was summarized in TreeAnnotator. Tracer 1.6 (Rambaut et al. 2014) was used to compare the tested tree models, to verify convergence of the runs and to perform Bayesian Skyride reconstruction (using the default settings) estimating the female effective population size (N_ef_). The output tree was visualized in FigTree 1.4.2 (available at http://tree.bio.ed.ac.uk/software/figtree/). Both the haplotype network and the phylogenetic tree were graphically edited in Inkscape 0.91 (available at https://inkscape.org/en/).

### MUTATION RATE

We took advantage of the known evolutionary history and the star‐like pattern of haplotypes to estimate the mutation rate in the Wrangel Island samples. Simulations were performed using *fastsimcoal ver. 2.5.2.2* (Excoffier et al. 2013) and the number of haplotypes was estimated with *arlsumstats ver. 3.5.2* (Excoffier and Lischer 2010), both controlled by custom R scripts (R Development Core Team 2013). We assumed that the population on Wrangel Island can be modeled as an isolated and continuous population from the time of the bottleneck (∼12 kyr calBP) to the time of extinction (∼4 kyr calBP). We performed coalescent simulations on a 100 × 100 grid composed of values from a range of constant female effective population sizes (N_ef_: 1 – 10,000,000 individuals) and mutation rates (μ: 0.07 – 667 × 10^−8^ site^−1^ year^−1^, corresponding to a range 0.1 – 1000 × 10^−7^ site^−1^ generation^−1^) both on an equally spaced log‐scale. For each combination of parameters we performed 1000 simulations of a DNA fragment of the same size as our alignment (16,506 bp), forcing all lineages to coalesce into one at the time of isolation so that only one haplotype would be present in the founder population of the island. In each simulation, we sampled individuals at the same time as the mean calibrated age of our samples (Table 1) and the probability of observing exactly seven haplotypes (as inferred from the haplotype network; see the Results) in the simulated Wrangel samples was reported. The female generation time was set to 15 years (Palkopoulou et al. 2013). Coalescent simulations were run both assuming no transition bias (0.33), and a high transition bias (0.98, as in Palkopoulou et al. 2013) with no marked effect in the estimated mutation rates.

## Results

### MITOCHONDRIAL HAPLOTYPES

**Figure 2:**
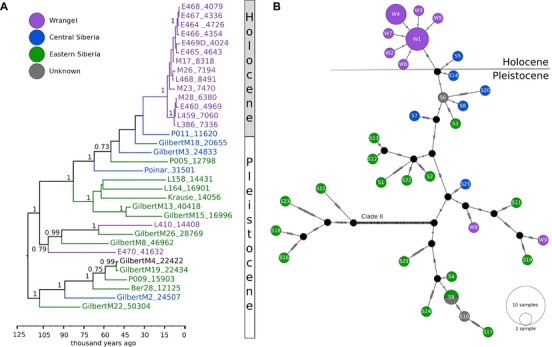
A Bayesian Phylogeny of clade I mammoths with finite radiocarbon dates (A) and median‐joining haplotype network of all 42 Pleistocene and Holocene mammoths (B; W = Wrangel, S = Siberia). In the phylogeny, nodes with posterior probabilities above 0.7 are shown and numbers in the sample names indicate age.

Across the 16,506 base pairs (bp) of the mitochondrial genome, mammoths in our study were assigned to 34 unique haplotypes. The haplotype median‐joining network (Fig. 2B) clearly differentiated mammoths belonging to clades I and II, with Wrangel Island mammoths nested within clade I as shown previously (Nyström et al. 2010; Palkopoulou et al. 2013).

In the Holocene Wrangel population, we found seven mitochondrial haplotypes that formed a unique subgroup shaped in a star‐like pattern with multiple haplotypes surrounding what was presumably a single ancestral haplotype (W1, Fig. 2B). These seven haplotypes were not closely related to the haplotypes observed in Wrangel mammoths radiocarbon dated to the Late Pleistocene (E470, L410), nor did they show a close affinity to the end‐Pleistocene haplotypes from Chukotka, which is the geographically closest mainland region. Instead, the Holocene Wrangel Island haplotypes were most closely related to haplotypes observed in mammoths from Central Siberia (Fig. 2B; Table 1). The latter were also basal to the Holocene Wrangel mammoths in the phylogeny (Fig. 2A).

The Holocene Wrangel mammoths differed from all other mammoths by three unique mutations: one mutation in a region coding for transfer RNA (tRNA) valine and two mutations in protein‐coding genes, a synonymous mutation in *ND6* and a nonsynonymous mutation in *ATP6* (G457A).

#### Bayesian phylogenies and effective population size

The phylogeny inferred using Bayesian Skyride model (Fig. 2A) indicated a differentiation among Late Pleistocene and Holocene mammoths. While the Late Pleistocene mammoths generally formed long and well‐supported branches (posterior probability ≥0.95), Holocene Wrangel mammoths clustered into a monophyletic group with unresolved internal relationships and short branches, indicative of a rapid diversification.

**Figure 3:**
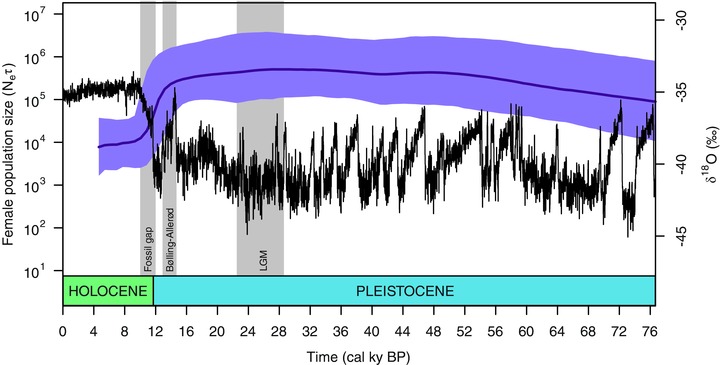
Bayesian Skyride plot of the female effective population size (N_ef_) based on 34 clade I mammoth samples with finite radiocarbon dates. The left *y*‐axis and the solid purple line represent median values of N_ef_ with the purple area indicating the 95% highest posterior density. The *x*‐axis is in calendar years before present. N_ef_ is scaled by a factor of generation time assumed to be 15 years. The right *y*‐axis and the black line show the climate record from the North Greenland ice core (North Greenland Ice Core Project members 2004).

The Bayesian Skyride plot (Fig. 3) revealed a sharp decrease in effective population size starting at about 15 kyr calBP, which coincides with the beginning of the Bølling‐Allerød interstadial (14.7–12.9 kyr calBP). The steep decline continued through the Pleistocene/Holocene boundary until approximately 9 kyr calBP. Assuming an average generation time of 15 years (as in Palkopoulou et al. 2013), N_ef_ dropped 15‐fold from ca 26,000 individuals (95% highest posterior density (HPD): 105,000–3500) prior to Bølling‐Allerød (14.7 kyr calBP) to ca 1700 individuals (95% HPD: 9000–160) in the early Holocene (10 kyr calBP).

#### Mutation rate

The mutation rate of the mitogenomes estimated by tip calibration (Table S2) with a Skyride tree prior yielded a mean rate of 1.31 × 10^−8^ site^−1^ year^−1^ (95% HPD: 0.79–1.84 × 10^−8^ site^−1^ year^−1^), whereas using a constant size tree prior yielded a mean rate of 1.36 × 10^−8^ site^−1^ year^−1^ (95% HPD: 0.83 – 1.93 × 10^−8^ site^−1^ year^−1^). The rate estimated by Bayesian Skyline analyses was 1.22 × 10^−8^ site^−1^ year^−1^ (95% HPD: 0.66 – 1.75 × 10^−8^). The extent of temporal signal in the data was validated by the date‐randomization test, which showed that the 95% HPD of the substitution rate estimated from 20 randomized datasets (2.41 × 10^−14^ – 0.5 × 10^−8^) is outside the 95% HPD of the estimate from real data (0.79 – 1.84 × 10^−8^).

**Figure 4:**
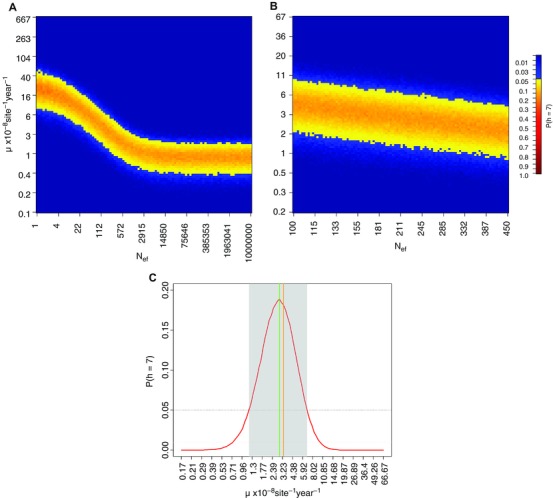
Probability of observing seven mitochondrial haplotypes in 14 Holocene Wrangel samples estimated from coalescent simulations using the mutation rate (μ) and the female effective population size (N_ef_) as exploratory parameters: (A) using wide priors, μ: 0.07–667 × 10^−8^ site^−1^ year^−1^ and N_ef_: 1–10,000,000 individuals; (B) using narrower priors better fitting the scenario of low effective population size on Wrangel Island (N_ef_: 100–450 individuals, and μ: 0.17–66.7 × 10^−8^ site^−1^ year^−1^); (C) summary of probabilities from simulations with narrower priors and incorporating all values of N_ef_ to account for uncertainty. The green line indicates the mutation rate corresponding with the highest probability. The orange line represents the estimated mutation rate using 328 individuals as effective population size as in (Palkopoulou et al. 2015) and assuming a 1:1 sex ratio (Nyström et al. 2010).

Coalescent simulations of the mutation rate of the Holocene Wrangel population suggested that prior knowledge about the population size is paramount for the estimation of the mutation rate. As depicted in Fig. 4A, the mutation rate estimate is stable and a little lower than the estimates obtained from BEAST for effective population sizes over 5000 females (∼0.9 × 10^−8^ site^−1^ year^−1^; Table S2), but it changes rapidly when lower effective population sizes are assumed. However, an effective size larger than 5000 females is rather unlikely for the Wrangel Island population, which was recently estimated to around 328 individuals for both sexes using genomic data (Palkopoulou et al. 2015). Moreover, it is unlikely that the effective population size exceed the carrying capacity of Wrangel Island, which has been estimated to between 149 and 819 individuals using Damuth's equation (Nyström et al. 2010). To more precisely estimate the mutation rate, assuming more realistic Wrangel population sizes and an 1:1 sex ratio (Nyström et al. 2010), we performed a second set of simulations with much narrower priors (N_ef_: 100–450 individuals, and μ: 0.17 – 66.7 × 10^−8^ site^−1^ year^−1^, Fig. 4B). The results from this second set of simulations indicated that the probability of observing seven Holocene Wrangel haplotypes was maximized when the mutation rate was 3.05 × 10^−8^ site^−1^ year^−1^ (95% HPD: 1.31 – 6.69 × 10^−8^ site^−1^ year^−1^; Fig. 4C).

## Discussion

### THE ORIGINS AND DEMOGRAPHY OF WRANGEL MAMMOTHS

The haplotype network showed signs of a highly diverse mammoth population throughout the Pleistocene. However, our results suggest that immediately after the isolation on Wrangel Island only one haplotype was present, based on the star‐like pattern of the haplotype network where all haplotypes are only one or two mutational steps from the modal haplotype. This conclusion is further supported by the results reported by Nyström et al. (2010) where only one mitochondrial haplotype was observed during the first 1500 years following the isolation of Wrangel Island. Reduction to a single maternal lineage is in agreement with mammoths going through a bottleneck and founder effect as they became isolated on Wrangel Island. The Wrangel Island's mammoth population was thus likely founded by a very limited number of females, presumably one herd considering that similarly to present elephant species mammoths likely formed matriarchal family groups. The matriarchal social structure could also explain why we observed a more pronounced founder effect using mitogenomes compared to previous results from nuclear data (Palkopoulou et al. 2015; Rogers and Slatkin 2017).

Interestingly, the samples genetically most similar to the Holocene Wrangel mammoths are also the ones geographically most distant, originating from Central Siberia, specifically from the Gydan Peninsula (GilbertM18), Taimyr Peninsula (P011, GilbertM3, Poinar), and New Siberian Islands (P005). The area comprising the Taimyr and Gydan Peninsulas is possibly the only region in Siberia with a continuous fossil record throughout the warm Bølling‐Allerød Interstadial when mammoths disappeared from other parts of Eurasia (Stuart 2005). During the Younger Dryas (YD; 12.9–11.7 kyr calBP), a colder period following the Bølling‐Allerød Interstadial, cold climate conditions allowed a reexpansion of the open steppe tundra habitat, which is thought to have enabled a reexpansion of the woolly mammoth from Taimyr to northwestern Siberia and northeastern Europe (Stuart 2005). Although speculative, these are the first genetic results supporting the hypothesis that the Taimyr Peninsula could have served as a source population for a Younger Dryas recolonization of northeastern Siberia, including what was later to become Wrangel Island.

#### Post‐LGM population size reduction and accelerated rate of evolution

The estimated female effective population size was rather stable during the Pleistocene and only started to decrease about 15 kyr calBP, at roughly the same time as the onset of the Bølling‐Allerød warming period and the time when mammoths disappeared from most of the Siberian mainland (Fig. 3). This decline in N_ef_ was approximately 15‐fold, which is comparable to the reduction observed in a previous study based on short mitochondrial sequences (Palkopoulou et al. 2013). The Bayesian Skyride analysis also indicated that the decline in effective population size continued until the final extinction, but at a considerably lower rate.

The coalescent simulations suggested a two‐ to threefold higher mutation rate in Holocene Wrangel Island samples (3.05 × 10^−8^ site^−1^ year^−1^; 95% HPD: 1.31–6.69 × 10^−8^ site^−1^ year^−1^), as compared to the estimates for all clade I mammoths from BEAST (1.31 × 10^−8^ site^−1^ year^−1^; 95% HPD: 0.79–1.84 × 10^−8^ site^−1^ year^−1^; Table S2). Although the HPDs are wide, these substitution rate estimates are lower than previously published rates based on a short hypervariable fragment of 741 bp (Barnes et al. 2007; Debruyne et al. 2008; Palkopoulou et al. 2013), but clearly higher than any other mutation rate published for mammoth or other proboscidean complete mitogenomes (Rohland et al. 2007). One possible explanation is that the higher substitution rate in the Wrangel Island population is associated with reduced purging of deleterious or slightly deleterious variants in the mitochondrial genome due to lower efficiency of purifying selection at long‐term low effective population sizes (Kimura 1957). This process potentially results in a higher than expected amount of polymorphism, and consequently an increased measurable rate of evolution. This is to our knowledge the first time that serially sampled data are used to show that the evolutionary rate changes in a species through time, as the species' population size decreases. These results consequently provide tentative support for expectations from the nearly neutral theory of molecular evolution (Ohta 1992). It should, however, be noted that a somewhat higher mutation rate could also result from a reduction in generation times in Wrangel Island mammoths as a consequence of insularity, as hypothesized by Rogers and Slatkin (2017). However, we find it unlikely that shorter generations would lead to an increase in evolutionary rate as high as two‐ to threefold (Nabholz et al. 2008; Bromham 2009).

#### Fixed mutations in the Wrangel population

Although the Holocene Wrangel mammoths formed a separate cluster genetically differentiated from the Pleistocene specimens, the Wrangel population was defined by only three synapomorphic mutations across the whole mitochondrial genome. Interestingly, the third mutation constituted a nonsynonymous substitution in the gene *ATP6* (G457A) encoding for subunit *a* of the ATP synthase enzyme, resulting in an alanine to threonine substitution at amino acid 157 (A157T), which had a derived state in all 14 Holocene Wrangel mitogenomes (Fig. S2). Mitochondrial ATP synthase is a key enzyme of the oxidative phosphorylation pathway and is responsible for ATP production in all living beings except for archaea (Vantourout et al. 2010). To assess genetic variation at the *ATP6* gene, we aligned the mammoth *ATP6* sequence with those of other taxa, randomly choosing one GenBank (Benson et al. 2013) sequence per species (Fig. S3) to stochastically capture variability in the dataset. We observed that the G457A mutation was located in a conserved part of the *ATP6* sequence and that no other species had a nonsynonymous mutation at that site (Fig. S3).

Assessing the potential fitness consequences of the A157T amino acid fixation is aided by the highly conserved structure of ATP6 across taxa. First, the location of this substitution is near an active site residue at position 159. Second, in humans, two different substitutions at the neighboring site 156 give rise to disease phenotypes. Both the L156R and L156P substitutions affect ATP synthase, resulting in a decreased proton flux and thereby decreased ATP synthase (Jonckheere et al. 2012). If we assume that these neighboring substitutions are a good proxy for the phenotypic effect of A157T, a reduced ATP synthase phenotype is possible (Cortes‐Hernandez et al. 2007).

One of the key predictions from the mutational meltdown theory (Lynch et al. 1995) is that deleterious mutations can become fixed in small populations due to strong genetic drift. Although an excess of deleterious mutations has previously been described in genome from a 4300‐year‐old Wrangel mammoth (Rogers and Slatkin 2017), our results from ATP6 might represent the first identification of a deleterious mutation that appears to have become fixed as a consequence of genetic drift during the establishment of the Wrangel Island population.

Rather than being the consequence of small effective population size for hundreds of generations (Rogers and Slatkin 2017), the excess of deleterious mutations in the Wrangel Island mammoth genome could be due to a severe bottleneck and founder effect at the time of the isolation, which may have also contributed to an increase in detrimental mutations immediately after the establishment of the population, as suggested by our results. Consequently, analyses of serially sampled genomic data from additional Wrangel Island mammoths are needed to resolve to what extent genetic drift during the Holocene led to a gradual accumulation of deleterious genetic variation.

Associate Editor: L. Bromham

## Supporting information

Fig. S1 A median‐joining haplotype network of the analyzed samples mapped against a clade II mammoth (GilbertM25; EU153453).

Fig. S2 Alignment of amino acid sequences of the ATP6 gene showing the nonsynonymous mutation fixed (purple) in all Holocene Wrangel mammoths (first 14 samples).

Fig. S3 Alignment of the ATP6 amino acid sequences of the woolly mammoth (*Mammuthus primigenius*) ‐ one Holocene Wrangel Island mammoth, Pleistocene clade I, II, and III mammoths, compared to various other mammals (with accession numbers in the sequence name), showing that the non‐synonymous mutation fixed in the Holocene Wrangel population cannot be found in any other species and is located within a rather conserved region.

Table S1 Support for three different tree model priors tested in the BEAST analysis – constant, Skyline, and Skyride – was estimated according to the path sampling and stepping‐stone sampling log marginal likelihoods. Bayes factors and the selected model are indicated.

Table S2 Mutation rate estimates for clade I mammoths calculated in BEAST.
